# The impact of preoperative anemia on postoperative anemia and related nutritional abnormalities after bariatric surgery: a multicenter cohort study

**DOI:** 10.3389/fnut.2025.1616340

**Published:** 2025-07-09

**Authors:** Yuntao Nie, An Ping, Baoyin Liu, Hua Meng

**Affiliations:** ^1^Department of General Surgery & Obesity and Metabolic Disease Center, China-Japan Friendship Hospital, Beijing, China; ^2^School of Basic Medical Sciences, Capital Medical University, Beijing, China; ^3^Department of Education, Beijing Luhe Hospital, Capital Medical University, Beijing, China

**Keywords:** anemia, bariatric surgery, nutritional deficiency, Iron deficiency, ferritin deficiency, vitamin B12 deficiency, folate deficiency

## Abstract

**Background:**

Anemia is a common nutritional complication after bariatric surgery, deteriorating quality of life. Despite 10–30% of patients with obesity being anemic preoperatively, current guidelines do not recommend managing anemia before surgery. This study aims to evaluate the impact of preoperative anemia on postoperative anemia and related nutritional abnormalities.

**Methods:**

Patients undergoing bariatric surgery at two bariatric centers from 2017 to 2021 were reviewed and followed for 1 year. Anemia was defined by WHO criteria. Nutritional abnormalities included ferritin, folate, and vitamin B12 deficiency, and low transferrin saturation (TS) levels.

**Results:**

A total of 452 patients were included, of whom 53 (11.7%) were diagnosed with anemia before surgery. Patients with preoperative anemia were more likely to develop postoperative anemia (69.8% vs. 34.8%, *p* < 0.001), ferritin deficiency (77.4% vs. 41.6%, *p* < 0.001), and low TS levels (56.6% vs. 20.3%, *p* < 0.001) than normal patients. Changes in hemoglobin, ferritin, folate, and vitamin B12 levels did not differ significantly between patients with preoperative anemia and normal patients. After fully adjusting for covariates, preoperative anemia was independently associated with postoperative anemia (OR 3.52, 95% CI 1.83–7.06, *p* < 0.001), moderate to severe anemia (OR 5.03, 95% CI 2.48–10.20, *p* < 0.001), ferritin deficiency (OR 3.77, 95% CI 1.74–8.17, *p* = 0.001), and low TS levels (OR 4.12, 95% CI 2.16–7.84, *p* < 0.001).

**Conclusion:**

Preoperative anemia significantly elevates the risk of postoperative anemia, especially moderate to severe cases, and increases the incidence of iron deficiency. It is crucial to actively screen for and correct preoperative anemia to enhance hemoglobin levels and prevent postoperative anemia.

## 1 Introduction

Anemia is a one of the most common nutritional complications after bariatric surgery, with an incidence rate of 10 to 40% (1, 2). Postoperative anemia can cause prolonged fatigue, weakness, malnutrition, extended recovery time, and reduced quality of life (3–5). In severe cases, it can lead to cognitive decline and hematologic disorders (3).

Clinicians usually focus on prophylactic nutrient supplementation postoperatively and treat anemia once it develops, often overlooking preoperative anemia correction. Although clinical guidelines commonly recommend comprehensive nutritional screening prior to bariatric surgery—including assessment of ferritin, vitamin B12, and folate levels—and advise correction of any deficiencies before surgery to potentially reduce the risk of anemia (6, 7), nearly 30% of patients still present with anemia preoperatively (8–10). Obesity-induced chronic inflammation increases the secretion of iron-regulating hormones like hepcidin, which inhibits iron absorption and utilization (11, 12). Additionally, the micronutrient and vitamin deficiencies common in patients with obesity, particularly iron and vitamin B12 deficiencies, further elevate the risk of anemia (13, 14). Substantial evidence has indicated that anatomical changes in the gastrointestinal tract after bariatric surgery make postoperative anemia challenging to manage with oral supplements alone (15–17). Its prevalence increases over time, highlighting the importance of anemia prevention (1, 18).

Previous studies have attempted to identify risk factors associated with anemia following bariatric surgery. It has been consistently observed that female patients, particularly premenopausal women, have a significantly higher incidence of postoperative anemia compared to males (19, 20). Preoperative hemoglobin level has also been identified as a key predictor, with higher baseline levels associated with a lower risk of postoperative anemia (21–23). Additionally, several small retrospective studies have suggested that preoperative anemia may be a risk factor for anemia after bariatric surgery, proposing that correction of anemia prior to surgery could potentially reduce postoperative incidence (19). However, in current clinical practice, preoperative correction of anemia remains uncommon, and existing clinical guidelines do not explicitly recommend it. The relationship between preoperative anemia and postoperative anemia or related nutrient deficiencies still warrants further investigation.

Therefore, this study aimed to explore the impact of preoperative anemia on postoperative anemia and related nutritional abnormalities in bariatric surgery patients.

## 2 Materials and methods

### 2.1 Study design and participants

We conducted a retrospective observational cohort study on all consecutive patients with obesity (body mass index [BMI] ≥ 27.5 kg/m^2^) who underwent sleeve gastrectomy (SG) or Roux-en-Y gastric bypass (RYGB) at two bariatric centers, specifically the China-Japan Friendship Hospital and Beijing Fuxing Hospital, from September 2017 to December 2021. It was registered on ClinicalTrials.gov (NCT06218953). All patients met the bariatric surgery indications defined by the Chinese Society for Metabolic & Bariatric Surgery (CSMBS) (24). Exclusion criteria were: (1) baseline renal failure; (2) vegetarian diet; (3) postoperative bleeding (drop in hemoglobin > 30 g/L or confirmed blood loss requiring treatment); and (4) lack of 1-year follow-up data.

This study followed the Strengthening the Reporting of Observational Studies in Epidemiology—Nutritional Epidemiology (STROBE-nut) statement (25). It was conducted in accordance with the Helsinki Declaration and approved by the Institutional Review Board (IRB) of China-Japan Friendship Hospital (2021-112-K70). Informed consent was waived by the IRB as the study was observational and noninvasive.

### 2.2 Data collection

Two investigators collected all data online from the electronic medical record system. Sociodemographic data included sex, age, height, weight, BMI, waist circumference, hip circumference, smoking history, alcohol consumption history, hypertension, systolic blood pressure, diastolic blood pressure, type 2 diabetes mellitus (T2DM), hyperuricemia, hypercholesterolemia, hypertriglyceridemia, and surgical procedures. Hypertension was diagnosed based on systolic blood pressure ≥ 140 mmHg, diastolic blood pressure ≥ 90 mmHg, or a history of hypertension (26). T2DM was diagnosed according to the American Diabetes Association guidelines (27): fasting plasma glucose (FPG) ≥ 7.0 mmol/L, 2-h plasma glucose ≥ 11.1 mmol/L during an oral glucose tolerance test, glycated hemoglobin (HbA1c) ≥ 6.5%, classic symptoms of hyperglycemia or hyperglycemic crisis, random plasma glucose ≥ 11.1 mmol/L, or a past diagnosis of T2DM. Hyperuricemia was diagnosed based on fasting uric acid levels, measured twice on the same day under a normal purine diet: > 420 μmol/L in males and > 357 μmol/L in females (10). Hypercholesterolemia was diagnosed based on total plasma cholesterol > 5.17 mmol/L. Hypertriglyceridemia was diagnosed based on plasma triglycerides > 2.3 mmol/L (28).

All patients underwent physical examinations—including measurements of height, weight, waist circumference, and hip circumference—using standardized procedures recommended by the World Health Organization (WHO) (29). All measurements were performed on the morning of the surgery while the patients were in a fasting state, barefoot, and wearing light clothing. Weight was measured to the nearest 0.1 kg, and height was measured to 1 decimal point. Waist circumference was measured at the midpoint between the lower margin of the last palpable rib and the top of the iliac crest, while hip circumference was measured at the widest portion of the buttocks. The measuring tape was kept parallel to the floor. During both measurements, patients stood with feet together, arms relaxed at their sides, and body weight evenly distributed. Measurements were taken at the end of a normal expiration. BMI was defined as weight (kg) divided by height squared (m^2^). Waist-hip ratio (WHR) was calculated as waist circumference divided by hip circumference.

Laboratory measurement of blood samples was conducted at preoperative baseline (within 1 week before surgery) and at 1, 3, 6, and 12 months postoperatively. Due to the wide geographic distribution of the patients, there was a high rate of loss to follow-up for laboratory results at postoperative time points other than the 1-year mark. Therefore, subsequent analyses included only data from the preoperative and 1-year postoperative time points. The parameters measured included hemoglobin, ferritin, folate, vitamin B12, serum iron, and transferrin saturation (TS). Hemoglobin levels were measured by the sodium dodecyl sulfate method. Serum folate, vitamin B12, and ferritin levels were measured using chemiluminescent enzyme immunoassay. Serum iron was determined by ferrozine colorimetry method. TS refers to the ratio of serum iron to TIBC.

Weight loss 1 year postoperatively was expressed using BMI, ΔBMI, and % excess weight loss (%EWL) (30). ΔBMI was calculated by subtracting the postoperative BMI at 1 year from the initial BMI. %EWL was calculated as (initial weight − weight at 1 year postoperatively) / (initial weight − ideal weight) × 100.

### 2.3 Definition of anemia and nutritional abnormalities

Anemia was defined according to WHO criteria (31): hemoglobin <130 g/L for males and <120 g/L for females. The severity of anemia was classified into three categories: mild (hemoglobin ≥110 g/L), moderate (80 g/L ≤ hemoglobin <110 g/L), and severe (hemoglobin <80 g/L). Folate and vitamin B12 deficiencies were defined as serum levels < 10 nmol/L and < 150 pmol/L, respectively (32). Ferritin deficiency was defined as serum levels < 30 ng/mL, and low TS levels were defined as TS < 20% (33).

### 2.4 Surgical procedures

Symmetric three-port laparoscopic SG and RYGB were performed as described in our previous studies (34, 35). For laparoscopic SG, a linear stapler was used to resect the stomach, starting 4–6 cm proximal to the pylorus and continuing along a 36 Fr bougie to 1 cm from the gastroesophageal junction, followed by staple line reinforcement with sutures. For laparoscopic RYGB, a standard 30 mL gastric pouch was created, along with a 100 cm biliopancreatic limb and a 100 cm Roux limb. The gastrojejunostomy and jejunojejunostomy were constructed using a linear stapler and closed with hand-sewn sutures. Patients with long-duration T2DM, weakened islet function, or severe gastroesophageal reflux disease symptoms were recommended RYGB as a priority, but patient preference was also considered.

### 2.5 Postoperative nutritional supplementation

All patients followed a very low-calorie diet for 1 month postoperatively, consisting of one to three meal replacements per day supplemented with protein powder (1.0–1.2 g/kg/day of protein). Each meal replacement contains 110 μg of folate, 0.8 mg of vitamin B1, 1.2 mg of vitamin B6, 1.5 μg of vitamin B12, 40 mg of vitamin C, 420 mg of calcium, and 8 mg of iron. After the first month, a personalized dietary plan was developed by a clinical dietitian (Z. W.). Patients were advised to take two tablets per day of an oral vitamin/micronutrient supplement, which contains nutrients in doses consistent with the American Society for Metabolic and Bariatric Surgery (ASMBS) guidelines (6): 45 mg of iron, 800 μg of folate, 1,000 μg of vitamin B12, 3,000 IU of vitamin D, 4 mg of vitamin B6, 50 mg of magnesium, and 170 mg of calcium. At follow-up, patients diagnosed with iron deficiency were prescribed oral iron supplementation at a daily dose of 150–200 mg. For those who did not respond to or could not tolerate oral iron, parenteral iron therapy was administered as an alternative. This supplementation regimen was recommended for lifelong use in both SG and RYGB patients.

### 2.6 Statistical analysis

Continuous variables were presented as mean ± standard deviation or median and interquartile range, while categorical variables were presented as frequency and percentage. Continuous variables between two groups were compared using the Student t-test or Wilcoxon rank-sum test, depending on data normality. Categorical variables were compared using the Chi-square test or Fisher’s exact test. Baseline and 1-year laboratory variables were compared using paired sample t-tests or Wilcoxon rank-sum tests. Changes from baseline between the two groups were analyzed using analysis of covariance (ANCOVA), adjusting for baseline laboratory variables, gender, age, BMI, waist circumference, hip circumference, surgical procedure, smoking history, alcohol consumption, hypertension, T2DM, hyperuricemia, hypercholesterolemia, and hypertriglyceridemia. Multivariable logistic regression models were used to explore the relationship between anemia and related nutritional abnormalities and preoperative anemia. Three models were established: Model 1 without adjustment, Model 2 adjusted for sex, age, and BMI, and Model 3 further adjusted for waist circumference, hip circumference, surgical procedure, smoking history, alcohol consumption, hypertension, T2DM, hyperuricemia, hypercholesterolemia, and hypertriglyceridemia. *p*-values were two-tailed, with statistical significance considered at *p* < 0.05. All statistical analyses were performed using Stata/MP 17.0 and R software (version 4.1.2).

## 3 Results

### 3.1 Patient characteristics

**Figure 1:**
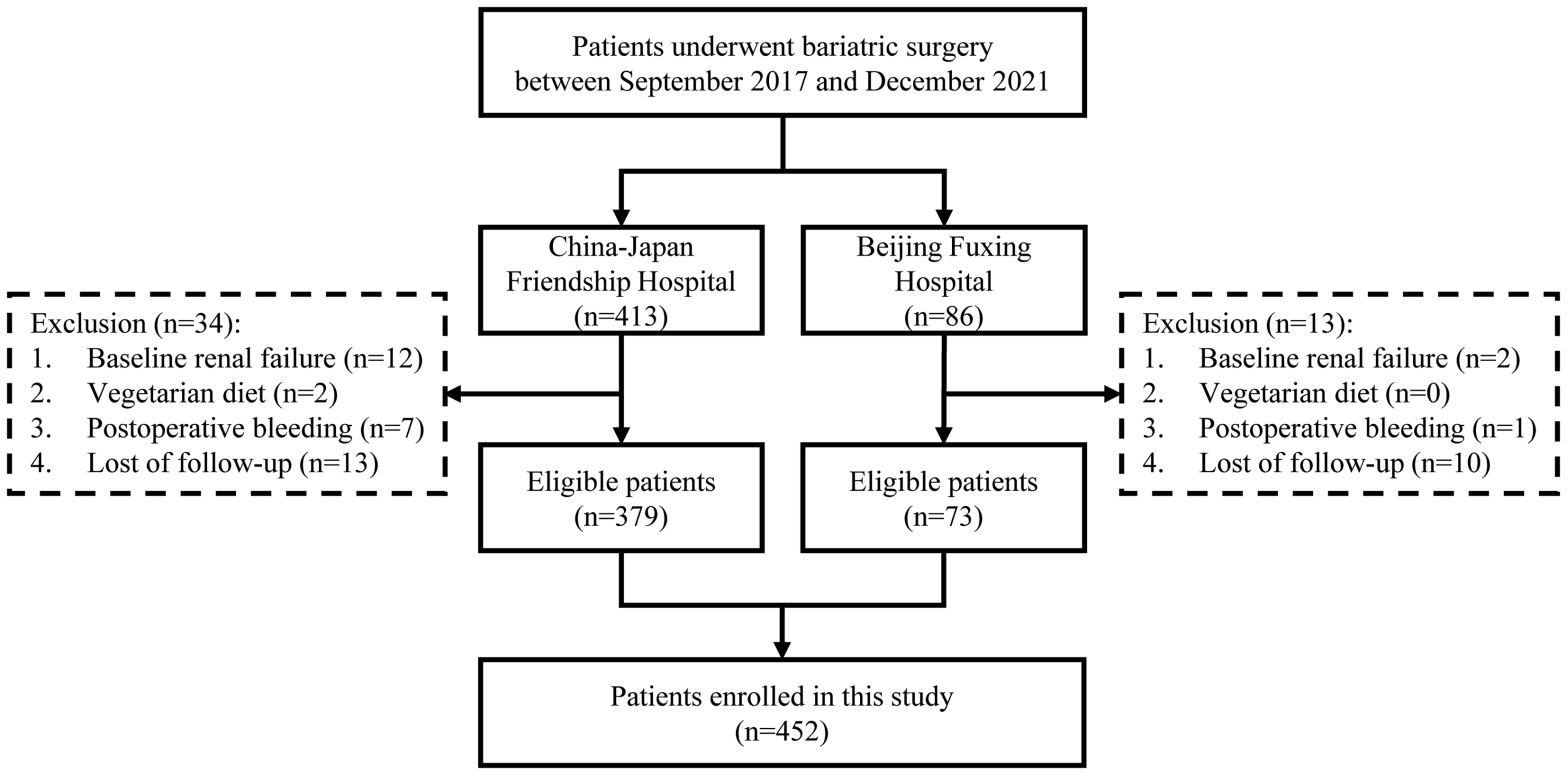
Flowchart of the study.

The study flow is illustrated in Figure 1. Table 1 shows the baseline characteristics of the patients. A total of 452 patients were included, with 53 (11.7%) diagnosed with preoperative anemia according to WHO criteria. Of all patients, 30.5% were male, with a mean age of 37.1 years and a median BMI of 37.1 kg/m^2^. Nearly 80% of the patients underwent SG, while the rest underwent RYGB. Patients with preoperative anemia were more likely to be female, non-smokers, and had a lower incidence of hyperuricemia compared to non-anemic patients.

**Table 1:** Baseline characteristics and weight loss after 1 year.

Variable	Overall (*N* = 452)	Normal (*N* = 399)	Preoperative anemia (*N* = 53)	*p*
Male, %	138 (30.5)	134 (33.6)	4 (7.5)	<0.001
Age (years), mean±SD	37.1 ± 10.7	36.9 ± 10.7	38.7 ± 10.1	0.242
Height (cm), mean±SD	168.0 ± 8.4	168.3 ± 8.6	165.7 ± 6.4	0.036
Weight (kg), median (IQR)	103.0 (90.0, 120.0)	104.0 (90.0, 120.5)	100.0 (91.0, 112.0)	0.319
BMI (kg/m^2^), median (IQR)	37.1 (32.8, 41.4)	37.2 (32.9, 41.5)	36.73 (32.6, 41.0)	0.894
WC (cm), mean±SD	117.1 ± 15.6	117.1 ± 15.8	117.1 ± 14.3	0.989
HC (cm), mean±SD	121.0 ± 14.2	120.9 ± 14.3	121.4 ± 14.1	0.797
WHR, mean±SD	1.0 ± 0.1	1.0 ± 0.1	1.0 ± 0.1	0.651
Surgical procedure, %				
SG	361 (79.9)	318 (79.7)	43 (81.1)	0.950
RYGB	91 (20.1)	81 (20.3)	10 (18.9)	
Smoking history, %	79 (17.5)	76 (19.0)	3 (5.7)	0.027
Alcohol consumption, %	67 (14.8)	60 (15.0)	7 (13.2)	0.883
Hypertension, %	212 (46.9)	187 (46.9)	25 (47.2)	1.00
SBP (mmHg), mean±SD	137.0 ± 18.9	137.4 ± 19.4	134.1 ± 14.7	0.237
DBP (mmHg), mean±SD	86.4 ± 14.6	86.8 ± 15.0	83.0 ± 10.6	0.080
T2DM, %	268 (59.3)	240 (60.2)	28 (52.8)	0.384
Hyperuricemia, %	281 (62.2)	261 (65.4)	20 (37.7)	<0.001
Hypercholesterolemia, %	156 (34.5)	143 (35.8)	13 (24.5)	0.141
Hypertriglyceridemia, %	111 (24.6)	101 (25.3)	10 (18.9)	0.393
BMI at 1 year, median (IQR)	26.4 (24.0, 29.4)	26.3 (23.9, 29.3)	27.4 (25.0, 29.5)	0.127
ΔBMI at 1 year, median (IQR)	10.2 (7.3, 13.5)	10.3 (7.3, 13.6)	9.1 (6.7, 12.1)	0.108
%EWL at 1 year, mean±SD	94.2 ± 37.8	95.1 ± 38.4	87.4 ± 32.8	0.163

Categorical data are reported as N (%), with *p* values from Chi-square test or Fisher’s exact test. Continuous data are presented as mean±SD or median (IQR), with *p* values from Student t-test or Mann–Whitney U test. *p* values are significant at *p* < 0.05. BMI, body mass index; DBP, diastolic blood pressure; EWL, excessive weight loss; HC, hip circumference; RYGB, Roux-en-Y gastric bypass; SBP, systolic blood pressure; SG, sleeve gastrectomy; T2DM, type 2 diabetes mellitus; WC, waist circumference; WHR, waist-hip ratio.

### 3.2 Weight loss after surgery

The median BMI 1 year postoperatively was 26.4 (24.0, 29.4) kg/m^2^, with a median ΔBMI of 10.2 (7.3, 13.5) kg/m^2^, and a mean %EWL of 94.2 ± 37.8%. No statistically significant differences in BMI (27.4 [25.0, 29.5] kg/m^2^ vs. 26.3 [23.9, 29.3] kg/m^2^, *p* = 0.127), ΔBMI (9.1 [6.7, 12.1] kg/m^2^ vs. 10.3 [7.3, 13.6] kg/m^2^, *p* = 0.108), and %EWL (87.4 ± 32.8% vs. 95.1 ± 38.4%, *p* = 0.163) were observed at 1 year between patients with and without preoperative anemia.

### 3.3 Hematological and nutrient parameters

**Figure 2:**
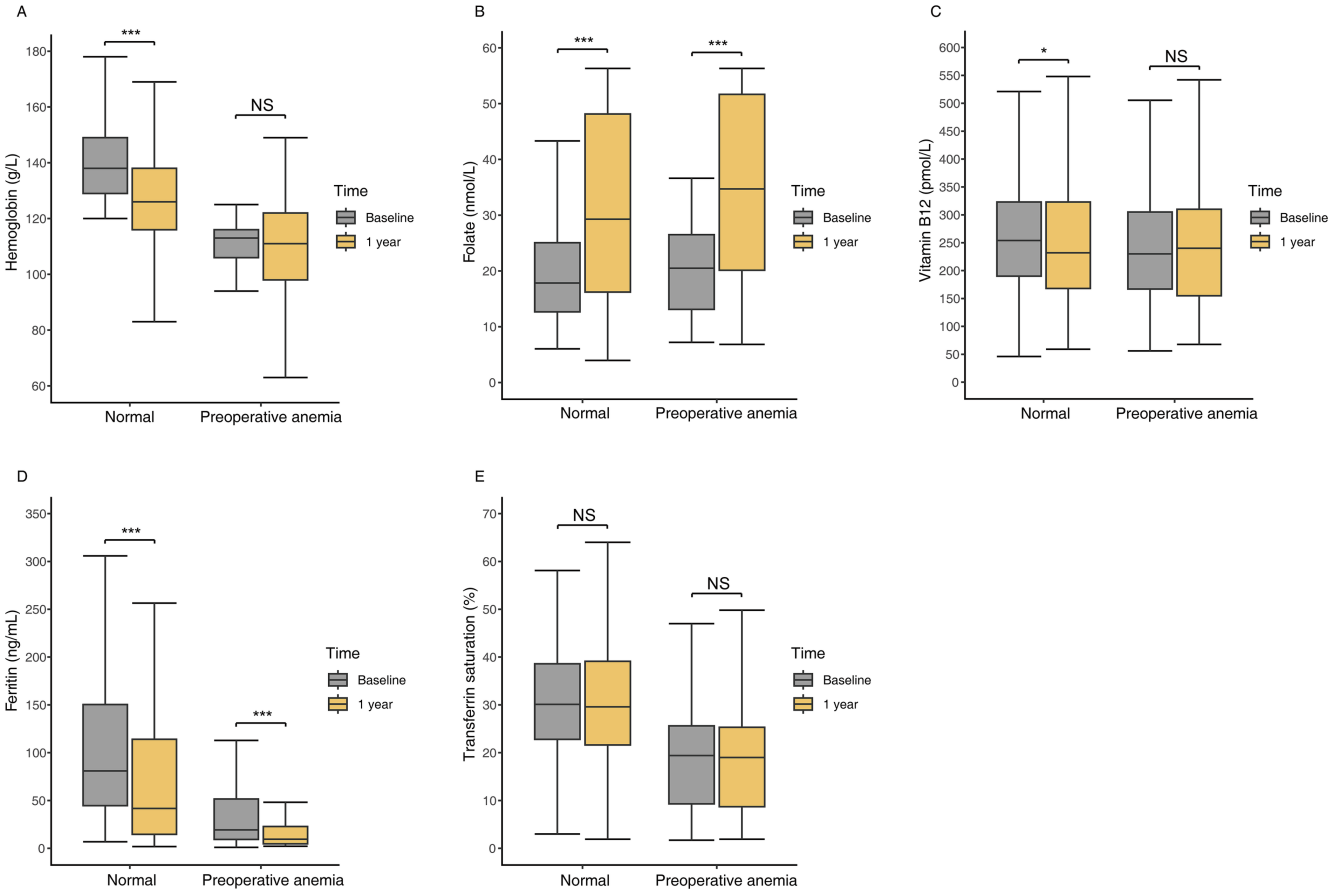
Hematologic and nutritional parameters at baseline and one year postoperatively in patients with preoperative anemia compared to those without anemia. **(A)** Hemoglobin levels. **(B)** Folate levels. **(C)** Vitamin B12 levels. **(D)** Ferritin levels. **(E)** Transferrin saturation levels. *: *p* < 0.05, **: *p* < 0.01, ***: *p* < 0.001, NS: no significant.

Patients with preoperative anemia had significantly lower baseline levels of hemoglobin, ferritin, and TS compared to non-anemic patients, while serum folate and vitamin B12 levels were similar (Table 2). At 1 year postoperatively, non-anemic preoperative patients showed significant decreases in hemoglobin, vitamin B12, and ferritin levels, and a significant increase in folate levels (Figure 2). Among preoperative anemic patients, ferritin levels significantly decreased, folate levels significantly increased, but there were no significant changes in hemoglobin, vitamin B12, and TS levels. The changes in hemoglobin, folate, vitamin B12, and ferritin levels at one year postoperatively did not differ significantly between the two groups. However, the decrease in TS was slightly greater in non-anemic preoperative patients compared to those with preoperative anemia (Table 2).

**Table 2:** Baseline and 1-year follow-up variables.

Variable	Normal (*N* = 399)	Preoperative anemia (*N* = 53)	*p*
Hemoglobin (g/L), mean±SD			
At baseline	139.8 ± 12.9	110.9 ± 7.3	<0.001
At 1 year	126.5 ± 18.4	108.7 ± 19.9	<0.001
Change from baseline	−13.3 ± 15.2	−2.2 ± 20.0	0.747
Folate (nmol/L), median (IQR)			
At baseline	17.8 (12.7, 25.0)	20.5 (13.1, 26.5)	0.449
At 1 year	29.3 (16.2, 48.1)	34.7 (20.1, 51.7)	0.076
Change from baseline	8.2 (−1.0, 19.8)	14.1 (3.4, 26.7)	0.411
Vitamin B12 (pmol/L), median (IQR)			
At baseline	259.0 (191.5, 338.5)	235.0 (174.5, 314.0)	0.260
At 1 year	235.0 (170.3, 337.1)	269.7 (162.0, 333.0)	0.497
Change from baseline	−11.0 (−89.9, 60.0)	14.0 (−67.1, 93.0)	0.309
Ferritin (ng/mL), median (IQR)			
At baseline	85.4 (47.3, 170.2)	19.3 (9.3, 51.5)	<0.001
At 1 year	43.6 (15.2, 118.4)	9.5 (4.7, 22.8)	<0.001
Change from baseline	−59.2 (−139.0, −15.0)	3.2 (−10.1, 27.8)	0.271
Transferrin saturation (%), mean±SD			
At baseline	31.4 ± 11.9	18.8 ± 12.2	<0.001
At 1 year	30.6 ± 14.3	18.5 ± 11.4	<0.001
Change from baseline	−0.8 ± 14.8	−0.3 ± 16.4	0.003

Baseline and 1-year postoperative parameters between two groups were compared by Student t-test or the Mann–Whitney U test. Change from baseline between two groups were compared by analysis of covariance, adjusting for baseline levels, sex, age, BMI, waist circumference, hip circumference, surgical procedure, smoking history, alcohol consumption, hypertension, T2DM, hyperuricemia, hypercholesterolemia, and hypertriglyceridemia.

### 3.4 Anemia and related nutritional abnormalities

**Figure 3:**
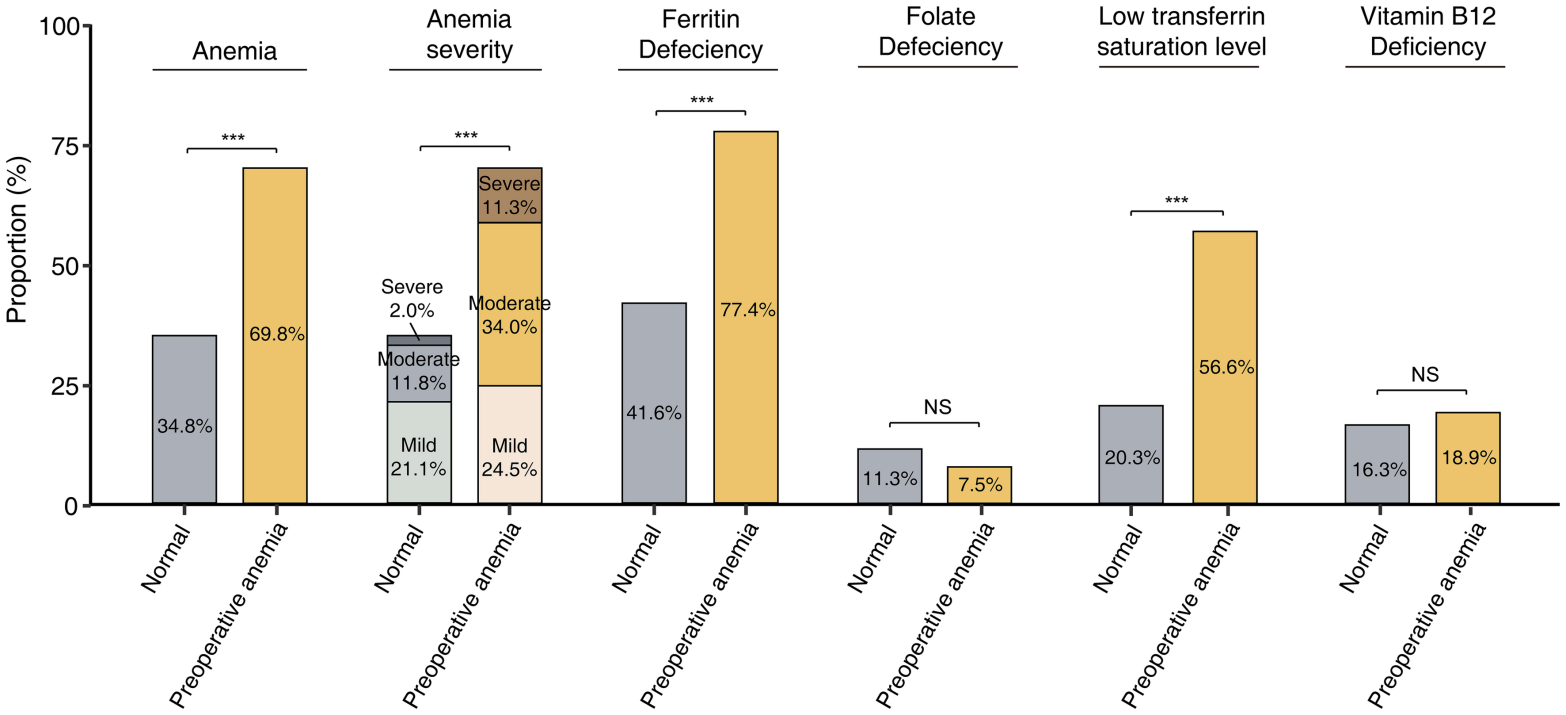
Incidence of postoperative anemia and related nutrient deficiencies in patients with and without preoperative anemia. *: *p* < 0.05, **: *p* < 0.01, ***: *p* < 0.001, NS: no significant.

At 1 year postoperatively, 37 of 53 (69.8%) preoperative anemic patients developed anemia, compared to 139 of 399 (34.8%) patients with normal preoperative hemoglobin levels (*p* < 0.001) (Figure 3). Among preoperative anemic patients, the incidence rates of postoperative mild, moderate, and severe anemia were 24.5, 34.0, and 11.3%, respectively, significantly higher than the rates of 21.1, 11.8, and 2.0% in patients with normal preoperative hemoglobin levels (*p* < 0.001). Compared to non-anemic patients, preoperative anemic patients had a significantly higher incidence of ferritin deficiency (77.4% vs. 41.6%, *p* < 0.001) and low TS levels (56.6% vs. 20.3%, *p* < 0.001). There were no significant differences in the incidence of folate deficiency (7.5% vs. 11.3%, *p* = 0.412) and vitamin B12 deficiency (18.9% vs. 16.3%, *p* = 0.636) between the two groups.

### 3.5 Association of preoperative anemia with anemia and related abnormalities

Table 3 shows the results of the multivariable logistic regression analysis. In Model 1, without covariate adjustment, preoperative anemia was significantly associated with an increased risk of postoperative anemia (odds ratio [OR] 4.33, 95% confidence interval [CI] 2.36–8.25, *p* < 0.001), particularly moderate to severe anemia (OR 5.18, 95% CI 2.81–9.54, *p* < 0.001). Additionally, preoperative anemia was significantly correlated with ferritin deficiency (OR 4.80, 95% CI 2.45–9.40, *p* < 0.001) and low TS levels (OR 5.12, 95% CI 2.82–9.29, *p* < 0.001), while no significant associations were observed with folate deficiency (OR 0.64, 95% CI 0.19–1.67, *p* = 0.415) or vitamin B12 deficiency (OR 1.19, 95% CI 0.57–2.50, *p* = 0.636). Model 2 showed similar findings. In the fully adjusted Model 3, preoperative anemia remained significantly associated with a higher likelihood of postoperative anemia (OR 3.52, 95% CI 1.83–7.06, *p* < 0.001), including moderate to severe cases (OR 5.03, 95% CI 2.48–10.20, *p* < 0.001). Furthermore, preoperative anemia was strongly linked to an elevated risk of ferritin deficiency (OR 3.77, 95% CI 1.74–8.17, *p* = 0.001) and low TS levels (OR 4.12, 95% CI 2.16–7.84, *p* < 0.001). However, no significant association was found between preoperative anemia and the risks of folate deficiency (OR 1.08, 95% CI 0.29–3.95, *p* = 0.909) or vitamin B12 deficiency (OR 1.40, 95% CI 0.60–3.28, *p* = 0.439).

**Table 3:** Unadjusted and adjusted ORs for anemia and related abnormalities by preoperative anemia status.

Abnormality	Model 1		Model 2		Model 3	
	OR (95% CI)	*p*	OR (95% CI)	*p*	OR (95% CI)	*p*
Anemia						
Normal	1.00 (reference)		1.00 (reference)		1.00 (reference)	
Preoperative anemia	4.33 (2.36–8.25)	<0.001	3.38 (1.82–6.53)	<0.001	3.52 (1.83–7.06)	<0.001
Moderate to severe anemia						
Normal	1.00 (reference)		1.00 (reference)		1.00 (reference)	
Preoperative anemia	5.18 (2.81–9.54)	<0.001	4.22 (2.24–7.95)	<0.001	5.03 (2.48–10.20)	<0.001
Folate deficiency						
Normal	1.00 (reference)		1.00 (reference)		1.00 (reference)	
Preoperative anemia	0.64 (0.19–1.67)	0.415	1.00 (0.29–3.39)	0.997	1.08 (0.29–3.95)	0.909
Vitamin B12 deficiency						
Normal	1.00 (reference)		1.00 (reference)		1.00 (reference)	
Preoperative anemia	1.19 (0.57–2.50)	0.636	1.51 (0.70–3.24)	0.294	1.40 (0.60–3.28)	0.439
Ferritin deficiency						
Normal	1.00 (reference)		1.00 (reference)		1.00 (reference)	
Preoperative anemia	4.80 (2.45–9.40)	<0.001	3.43 (1.64–7.17)	0.001	3.77 (1.74–8.17)	0.001
Low transferrin saturation level						
Normal	1.00 (reference)		1.00 (reference)		1.00 (reference)	
Preoperative anemia	5.12 (2.82–9.29)	<0.001	4.03 (2.19–7.44)	<0.001	4.12 (2.16–7.84)	<0.001

Model 1: unadjusted. Model 2: adjusted for sex, age, and BMI. Model 3: adjusted for sex, age, BMI, waist circumference, hip circumference, surgical procedure, smoking history, alcohol consumption, hypertension, T2DM, hyperuricemia, hypercholesterolemia, and hypertriglyceridemia. CI, confidential interval; OR, odds ratio.

## 4 Discussion

Anemia is a common comorbidity among patients with obesity (8–10), yet the necessity of correcting anemia before bariatric surgery remains debated. Our study found that preoperative anemia significantly increases the risk of postoperative anemia, particularly moderate to severe cases, and substantially raises the incidence of iron deficiency. Postoperative changes in hematological and nutritional parameters were similar between patients with and without preoperative anemia. Even after adjusting for covariates, preoperative anemia was still associated with a significantly higher risk of postoperative anemia and iron deficiency.

Chronic inflammation from obesity causes the liver to release inflammatory factors like TNF-α, IL-1, and IL-6, which upregulate hepatic hepcidin synthesis and may reduce erythropoietin synthesis and activity (12, 36). Hepcidin binds to ferroportin, promoting its internalization and degradation, inhibiting iron absorption in the intestine and causing iron sequestration in the liver and macrophages (12, 33, 36). Additionally, while obesity is often linked to excessive intake of high-calorie foods, imbalanced diets can lead to malnutrition, especially in vitamins and minerals (13, 14). Consequently, patients with obesity are at a higher risk of developing anemia. In our study, 11.7% of obese patients were anemic preoperatively, consistent with the reported prevalence of 10–30% in previous literature (9, 37, 38). Variations in the prevalence of anemia among patients with obesity across different studies are mainly attributable to dietary differences. Regions with insufficient red meat and organ intake tend to have higher anemia rates.

Our findings suggest that preoperative anemia significantly increases the incidence of postoperative anemia, consistent in multivariable models adjusted for all covariates. Previous studies have also focused on the association between lower preoperative hemoglobin levels and postoperative anemia after bariatric surgery. Research by Assakran et al. (21) in Saudi Arabia’s Qassim region reported a postoperative anemia prevalence of 28.1%, identifying low preoperative hemoglobin levels as an independent risk factor. Similarly, Pan et al. (23) reported comparable results, establishing a predictive model for iron deficiency anemia 1 year post-bariatric surgery, with preoperative hemoglobin levels as key predictors. Lee et al.’s study indicated that a preoperative hemoglobin level of 15.6 mg/dL is an important predictor for postoperative anemia (22). Our previous meta-analysis also suggested that preoperative anemia is a high-risk factor for both short-term and long-term anemia after sleeve gastrectomy (1).

In this study, the incidence of moderate to severe anemia was 17.5% (79/452). A 2022 study by Barzin et al. (39) reported similar findings and noted an increase in incidence over time. We also observed that preoperative anemic patients had a significantly higher incidence of postoperative moderate-to-severe anemia compared to those with normal preoperative hemoglobin levels (45.3% vs. 13.8%, *p* < 0.001). Given that moderate to severe anemia has more pronounced symptoms than mild anemia and is more likely to cause cognitive impairment, hematological disorders, and increased transfusion risk, preoperative anemia management should be more proactive.

Ferritin, a specific measure of iron storage, is commonly used with TS to diagnose iron deficiency, identified as the primary cause of postoperative anemia in previous literature (40–42). We found that preoperative anemic patients had significantly higher risks of ferritin deficiency and low TS levels 1 year postoperatively compared to those with normal preoperative hemoglobin levels. Despite lower initial iron stores in anemic patients, postoperative changes in ferritin levels showed no significant difference between the two groups, suggesting similar iron depletion trajectories. This can be attributed to factors like anatomical changes post-surgery, reduced gastric acid production, bypassing the proximal jejunum, and decreased red meat intake. These factors negatively impact iron absorption consistently in all bariatric surgery patients, regardless of preoperative anemia status. Consequently, higher preoperative iron stores may prevent a rapid decline in ferritin levels to deficiency postoperatively. Therefore, identifying and correcting iron deficiency before surgery is crucial, as it can delay the postoperative decline in ferritin levels, maintain higher iron levels, and prevent anemia.

Vitamin B12 and folate deficiencies can lead to megaloblastic anemia. While some studies found that these nutrient levels risk declining after bariatric surgery (43), we did not observe preoperative anemia exacerbating vitamin B12 and folate deficiencies. On one hand, liver stores of vitamin B12 are typically sufficient for several years, so deficiency may not manifest even years post-surgery (10). Additionally, we provide regular vitamin supplements postoperatively, reducing vitamin B12 deficiency in long-term follow-up. Moreover, folate absorption is mainly influenced by dietary choices. Given that the Chinese diet is predominantly grains and vegetables, severe folate deficiency is less common among our patients (8).

According to the British Obesity and Metabolic Surgery Society and ASMBS guidelines (6, 7, 44), postoperative prophylactic nutritional supplementation is routinely recommended to prevent iron, vitamin B12, and folate deficiencies. If deficiencies are identified during follow-up, enhanced supplementation is advised. However, adherence to these guidelines is often low (45), and even with recommended prophylactic supplementation, anemia incidence tends to increase over time (16). Unfortunately, standard oral iron supplementation is typically effective in maintaining iron stores but often ineffective in treating postoperative iron deficiency, as it can take 6–12 months to replenish iron stores (46, 47). Our study highlights the gap in existing guidelines for preoperative anemia management in bariatric patients, emphasizing the need for tailored recommendations in this population. All bariatric surgery patients should undergo thorough screening for preoperative anemia and potential insufficient iron storage. Preoperative iron therapy should be actively administered to correct anemia and prevent postoperative iron deficiency (48), particularly moderate to severe anemia.

This study has several limitations. First, as a retrospective study, it is subject to inevitable selection bias. Second, the study was conducted at two bariatric centers in China, so it is uncertain whether the conclusions can be generalized to Western countries with significantly different dietary patterns. Third, the follow-up period was only 1 year, limiting our ability to assess the long-term impact of preoperative anemia on hematologic parameters. Consequently, we plan to continue tracking these patients and conduct cohort studies with longer follow-up periods to evaluate long-term effects.

In conclusion, we revealed that preoperative anemia significantly increases the risk of postoperative anemia and iron deficiency in bariatric surgery patients. These findings highlight the critical need for preoperative anemia screening and correction to improve patient outcomes. Future bariatric surgery guidelines should incorporate these considerations to enhance patient care and reduce postoperative nutritional complications.

## Data Availability

The raw data supporting the conclusions of this article will be made available by the authors, without undue reservation.
